# Pilot Study of Ondansetron in Improvement of Pediatric Colonoscopy Preparation Outcomes at an Urban Academic Center

**DOI:** 10.1097/PG9.0000000000000366

**Published:** 2023-09-08

**Authors:** Dalia Arostegui, Paige Armaly, Kenny Castro Ochoa, Vivian Vega Lemus, Juveria Peshimam, Shagun Sharma, Steven Schwarz, Thomas Wallach

**Affiliations:** From the * Division of Pediatric Gastroenterology, Department of Pediatrics, SUNY Downstate Health Sciences University, Brooklyn, NY; † Department of Pediatrics, SUNY Downstate Health Sciences University, Brooklyn, NY.

**Keywords:** colonoscopy, Ondansetron, preparation

## Abstract

**Objectives:**

To gather initial data on the effectiveness and tolerability of the addition of Ondansetron to bowel preparation regimens to justify a funded, larger, placebo-controlled study.

**Methods:**

**Design, Setting, and Participants:**

A total of 41 pediatric and young adult (age 2–22) patients participated in a single center, open label, parallel randomized trial, with simple randomization. All patients were recruited as outpatients, and all procedures occurred as outpatient procedures, with both recruitment and procedures occurring at a low-resource urban academic medical center in Brooklyn.

**Interventions and Outcome Measures:**

The intervention studied was a single dose of oral-dissolving tablet Ondansetron provided before initiation of bowel preparation using a standardized prep of Polyethylene Glycol 3350 and Bisacodyl. There were 2 arms, a study arm using typical preparation (Polyethylene Glycol 3350 and Bisacodyl) and Ondansetron, and a control arm (Polyethylene Glycol 3350 and Bisacodyl). Patients received standard weight-based dosing. The primary outcome measure assessed was the Boston Bowel Preparation Scale (BBPS) to assess efficacy of preparation. Secondary objectives included evaluation of patient satisfaction via a survey answered by each patient. The questionnaire assessed the presence of the following symptoms during bowel prep: abdominal pain, nausea, bloating, vomiting, scale of ease/difficulty, and if the entire bowel prep was completed.

**Results:**

No benefit to BBPS from the addition of Ondansetron to bowel preparation was observed. Statistically significant improvement in reports of abdominal pain (35% decrease in Ondansetron arm) was noted with a *P* = 0.019. No statistically significant improvement was noted in other symptoms although all domains showed nonsignificant improvement in the Ondansetron arm.

**Conclusion:**

No benefit to efficacy of preparation as measured by the BBPS was observed. A single dose of Ondansetron before bowel preparation reduced reports of abdominal pain by 35%, with other symptomatic improvements suggesting possible improvements to be confirmed by a higher-powered study. Trial registration: NCT05439772.

**What Is Known**

- The most common reason for failed bowel preparation is poor tolerance, in particular challenges with nausea and vomiting secondary to the volume required for preparation.
- Poor bowel preparation increases risk of colonoscopy.
- Safe and well-tolerated antiemetic therapies exist.

**What Is New**

- This intervention had no observed impact on Boston Bowel Preparation Score in our pilot study.
- A single dose of Ondansetron before initiation of bowel prep significantly reduced reports of abdominal pain by 35%.

## INTRODUCTION

Colonoscopies are routinely performed in children to assess multiple gastrointestinal diseases. A bowel preparation (bowel prep) is required before any colonoscopy to allow for clear visualization and improved safety during the procedure (1). However, standard bowel preps are often poorly tolerated, which can result in an incomplete or slow, and thus less effective, bowel cleanout. It has been noted that suboptimal bowel preps can be seen in one-third of colonoscopies (1). While there are no standardized regimens, Polyethylene Glycol 3350 (often with other agents) has become the most popular to use in children (1). However, it is known that Polyethylene Glycol 3350 preparation has multiple side effects, with commonly noted issues including nausea, abdominal pain, and bloating (1–4). Some prior work has shown that suboptimal bowel prep is most often secondary to vomiting during preparation, with nausea being a major predictive factor of bowel prep failure (5). Although Polyethylene Glycol agents (with or without electrolytes) have been shown to be effective and well tolerated, a survey conducted by the North American Society for Pediatric Gastroenterology, Hepatology, and Nutrition, among the pediatric population patients frequently have difficulty with the volume required, resulting in cancelation or rescheduling of the procedure in 20% of cases (3). Other risk factors that have been found for poor bowel preparation include race, English as a second language, and Medicaid use (6,7).

In addition to cancelation and rescheduling of procedures, a suboptimal bowel prep may lead to missed diagnosis, difficulty with the procedure itself and diminished safety (5). As nausea and vomiting are heavy predictors of failure, therapy targeted at improving these symptoms may improve bowel prep tolerance. It is of particular importance and relevance to study this in at-risk patient populations, which per prior literature appear to be the most likely to suffer suboptimal preparation and resulting risks and delays in care (6,7).

Serotonin (5-hydroxytryptamine [5-HT]) is a major mediator in the gut that sends signals via afferent nerves to influence gut motility and secretion. Ondansetron is a 5-HT3 receptor antagonist that blocks the vagal stimulation induced by 5-HT4, which reduces gut motility and secretion and thus reducing nausea and vomiting (8). Ondansetron is frequently used in combination with oral rehydration therapy in children with vomiting secondary to acute gastroenteritis and has been found in multiple studies and 1 meta-analysis, to be highly safe and effective (8–12). While serotonin is involved in intestinal motility, and constipation has been reported as a side effect of Ondansetron in a study by Bryson (13) in 7% of patients, initial toxicity work noted diarrhea as a side effect in 9% of patients (14). Single dose studies of Ondansetron typically are performed in episodes of gastroenteritis, and actually demonstrate an association with increased diarrhea (15,16). Accordingly, in this study we set out to complete an open-label pilot trial in a historically at-risk (high Medicaid, high-English as a second language, high African American) population comparing the addition of 1 dose of Ondansetron before initiation of the bowel prep with our traditional bowel prep model of Polyethylene Glycol 3350 and Bisacodyl.

## METHODS

We completed a pilot study using a prospective, open label, parallel simple randomized design with a control and study group. Inclusion criteria included patients undergoing colonoscopy between the ages of 2–22. Exclusion criteria included known long QT or arrhythmia as Ondansetron may in theory cause prolongation of the QT interval, although it has been found to occur in patients with underlying heart conditions and found to be a dose dependent phenomenon not occurring at current recommended doses (11,17). Patients were enrolled and consent was obtained at the time that the clinical decision was made to complete a colonoscopy during clinic visits. Simple randomization was completed at that time by the enrolling physician via a coin flip (ie, heads: control group, tails: study group). All patients were informed that the trial medication addresses nausea during the consent process, but no other symptoms were discussed.

The control arm received our typical cleanout regimen including Polyethylene Glycol 3350 and Bisacodyl (chewable if unable to swallow tablets) and the Ondansetron arm received the same in addition to Ondansetron. Polyethylene Glycol and Bisacodyl dosages were determined by age group. Patients older than 10 years received 12 capfuls of polyethylene glycol in 64 ounces of water along with 3 bisacodyl tablets, ages 5–10 received 7 capfuls of polyethylene glycol in 30 ounces of water along with 2 bisacodyl tablets and ages <5 were given 5 capfuls of polyethylene glycol and 1 tablet of bisacodyl. Ondansetron dosage was determined by the weight of the patient. Patients <20 kg receiving 2 mg, >20 kg 4 mg, and >40 kg 8 mg, with only 1 dose administered before initiating Polyethylene Glycol 3350 consumption. Standard patient instructions are available in the Supplemental Data, Supplemental Digital Content, http://links.lww.com/PG9/A135. Neither patient nor investigators were blinded to the treatment arm and patients were aware of the addition of Ondansetron and informed that it is an antinausea medication. The procedures were performed at SUNY Downstate Medical Center and/or a satellite ambulatory center. RedCap was used to track data and allow for secure and easily interrogated results.

The primary objective of our study was to enhance the quality of colon visualization during colonoscopy. Our primary outcome measure was improvement in the Boston Bowel Preparation Scale (BBPS) (18). BBPS is a tool validated in adult populations that has previously been used for research in pediatric populations to score colonoscopy preparations (3). The BBPS score is a 4-point scoring system applied to each segment of the colon: right colon, transverse colon, and left colon. Each segment is assigned a score between 0 and 3 with 0 being an inadequately prepped segment and 3 being an excellent prep. The scores are added for a total score between 0 and 9 with 9 designating an entirely clean prep (19). Training sessions on the application of the BBPS were provided to participating endoscopists to decrease interobserver bias. Given the small size and limited resources of our urban hospital, and need for communication with patients, endoscopists were not able to be blinded to the treatment arm. As a pilot study with no prior data, sample size was based on volume sufficient to determine a 2.5-point improvement in BBPS (20/arm) using a 2-tailed *t* test with a power of 0.8 and *P* value of 0.05, based on the assumption that our baseline mirrored prior studies with a mean BBPS of 5.7 and improvement secondary to interventions of 2–3 points (12,13). IRB approval was obtained and oversight maintained by the SUNY Downstate Institutional Review Board. A clinical trial protocol was posted to clinicaltrials.gov.

The secondary objective was to evaluate patient tolerance via a survey answered by each patient. The questionnaire entails whether the patient had abdominal pain, nausea, bloating, vomiting, scale of ease to finish the colon prep, and if the entire colon prep was completed.

### Analysis

The primary outcome was defined as the mean score of the colon preparation given by BBPS score in the study arm (Ondansetron group) compared to the control arm. We compared mean BBPS scores between the study and control arms using student *t* test analysis with *P* value cutoff of 0.05. For the secondary outcome, we looked at binary questionnaire outcomes using a Fischer exact *t* test, 2-tailed test, with a *P* value cutoff of 0.05. Calculations were completed using Prism Graphpad software.

## RESULTS

Among 41 patients included in this pilot study, 20 were a part of the control group and 21 were a part of the study group. The demographics and characteristics are shown in Table 1. The median age was 14.4 years (range: 2–22, standard deviation 5.17). There were slightly more females (54%) than males (46%). The majority of participants self-identified as Black (66%, n = 27) followed by White (27%, n = 11) and Asian (7%, n = 3). A amount of 75% or greater of patients on both arms used Medicaid insurance.

**TABLE 1.** Demographics and characteristics of study participants

	Control (21)	Ondansetron (20)
Median age (IQR)	13 (2–19)	16.5 (9–22)
Sex assigned at birth		
Male	8 (38%)	11 (55%)
Female	13 (62%)	9 (45%)
Race		
Black	14 (67%)	13 (66%)
Caucasian	5 (24%)	6 (29%)
Asian	2 (9%)	1 (5%)
Presenting symptoms		
Abdominal pain	13 (62%)	13 (65%)
Diarrhea	6 (29%)	0 (0%)
Hematochezia	6 (29%)	6 (30%)
Constipation	3 (14%)	7 (35%)
FTT/weight loss	10 (48%)	4 (20%)
Vomiting	2 (10%)	1 (5%)
Medicaid insurance	17 (81%)	15 (75%)
IQR = inter-quartile range.		

In this pilot, 87% of all participants reported compliance with their bowel preps, including 90% of the study group and 86% of the control group. BBPS scores ranged from 0 to 9 with an average of 5.6 in the study group and 5.9 in the control group. The level of tolerability of the bowel prep, assessed as very easy to very difficult on a scale of 1–4, was 2.85 among the study group and 3 among the control group (*P* = 0.769).

**FIGURE 1.**
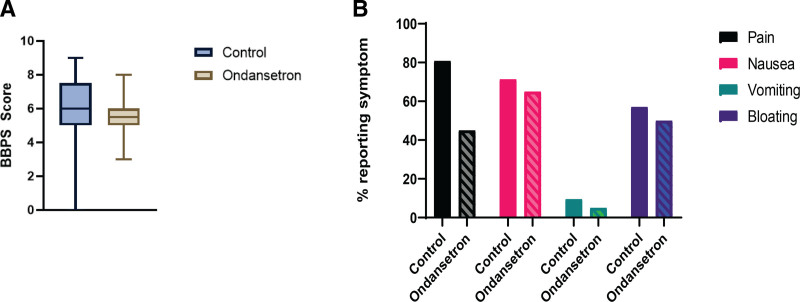
A) Chart comparing BBPS between control and study arms. Error lines represent standard error. B) Bar charts comparing symptom domain results by study arm. BBPS = Boston Bowel Preparation Scale.

Patient reported symptoms showed an improvement in abdominal pain associated with bowel prep in the Ondansetron arm (9/20 vs 17/21, absolute risk reduction 35%, *P* = 0.019). All other results showed improvement in symptom domains in the Ondansetron arm but did not reach significance (Fig. 1). As seen in our statistical analysis, the only symptom that was found to be significantly improved was abdominal pain (*P* = 0.025).

No negative side effects were reported. No complications or safety concerns were noted.

## DISCUSSION

Bowel preparation is a necessity for any colonoscopy, and in pediatric gastroenterology, poor tolerance of bowel prep is a persistent issue. The most frequently cited reasons for bowel prep failure are nausea, bloating, and pain (2). Our pilot study assessed the impact of Ondansetron, a selective 5-HT3 serotonin-receptor antagonist used clinically to ameliorate nausea, as an intervention to improve patient tolerance and comfort during prep in a minoritized and low-socioeconomic status (75%+ Medicaid insured) patient cohort. While our pilot study did not find a significant difference in BBPS scores representing cleanout quality, it is highly notable that we were able to demonstrate a statistically significant improvement in reports of abdominal pain (*P* = 0.025), as well as smaller improvements in multiple other symptom domains that may approach significance in a larger study.

Numerous studies have looked at ways to decrease abdominal pain with colonoscopy in adults (20,21), however our literature review has not demonstrated any prior studies assessing the impact of Ondansetron on pediatric tolerance of bowel prep. Our results demonstrating an improvement in abdominal pain during bowel prep are highly relevant to clinical practice, as current state practice has very limited tools to improve patient comfort during this necessary, unpleasant aspect of the procedure.

Our results fit well into current state of understanding of Ondansetron pharmacology. Alteration of vagal nerve serotonergic signaling can be linked to pain. Additional clinical research in other indications has also suggested the capacity for Ondansetron to relieve pain, such as Garsed et al’s demonstration of Ondansetron-mediated relief of abdominal pain in irritable bowel syndrome (22,23). Our secondary results in this study may be congruent with that finding, demonstrating benefit to abdominal pain through actions at 5-HT3 receptors on extrinsic sensory neurons that signal pain and discomfort.

There are several limitations in our study that need to be acknowledged. Firstly, our institution is resource-limited, and while we were able to incorporate an ideal at-risk patient population, we did not have the funding mechanism to institute a placebo-controlled RCT pilot. Second, our method had some limitations such as the absence of blinding for both researchers and participants, which could have led to biased responses. Researchers’ subjective assessment of bowel preparation using the Boston score may have also introduced some bias. Additionally, participants’ knowledge of their treatment group could have influenced their responses to the questionnaire. Furthermore, the sample size of the study was relatively small and the variation in indication for colonoscopy and age range may have had confounding effects on our data. Moreover, the uneven distribution of symptoms with randomization, particularly the notably larger fraction of patients in the Ondansetron study arm with constipation, may have also affected the impact of Ondansetron on preparation success. These limitations are inherent in a small pilot study completed at a small institution with relatively low colonoscopy volume, which limits our ability to generate statistically significant results. Nevertheless, our findings suggest that with a larger patient population, we may observe a difference in multiple symptom domains, and standardizing our study by indication for colonoscopy and age range could potentially demonstrate more clear benefit in terms of BBPS.

While our pilot study did not demonstrate improvement in bowel preparation, our secondary outcome measure did note an improvement in reports of abdominal pain. Our small sample size and the impact of simple randomization provides confounding elements that may not remain in a larger cohort. We were able to study the impact of this approach in a cohort generally not reached by research, in which 78% of our patient population utilized public insurance, precisely capturing groups that typically demonstrate suboptimal bowel preparation. We believe that our finding justifies further research with a larger population to determine both replicability of improvement in abdominal pain, and if other symptom domains improve in a larger, true placebo-controlled study.

## Supplementary Material


