# Supplementary material for: Pilot Study of Ondansetron in Improvement of Pediatric Colonoscopy Preparation Outcomes at an Urban Academic Center
Source: JPGN Rep. 2023 Sep 8;4(4):e366. doi: 10.1097/PG9.0000000000000366 (PMC10684247; doi:10.1097/PG9.0000000000000366)
Supplement: Supplementary file 1 [file pg9-4-e366-s001.pdf]

Please call the office if your child develops fever (101F or greater) or respiratory symptoms (coughing, difficulty breathing, nasal secretions, etc) within 48 hours prior to the procedure.
The number is:

718 270 4714

2-4 DAYS BEFORE THE PROCEDURE:

DATE:

Take 1 capful of miralax in 8oz of liquid two times a day. in addition, have additional 4 glasses of liquid daily.*

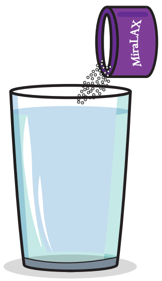

*fig. 1

1 DAY BEFORE THE PROCEDURE:

DATE:

A clear liquid diet should be started one day prior to the procedure. Please encourage your child to have something every 30-45 minutes. This will help to flush out the colon, keep your child well hydrated, and most importantly to your child, reduce the sensatjon of hunger.

The following is a list of items that are allowed:

- *Water*
- *Jell-O® (Avoid RED or PURPLE- colored drinks and Jell-O)*
- *Juice drinks without pulp*
- *Soda*
- *Water ice*
- *Popsicles*
- *Broth*
- *Sports drinks*
- *Pedialyte®*
- *Breast milk*

At Noon the day before the procedure, mix the 12 capful of Miralax in 64oz of clear liquids. Mix well. Drink 8-12 oz of Miralax mixture every 15-20 minutes until ALL 64oz ARE CONSUMED. It is very important that you drink all the Miralax mixture.*

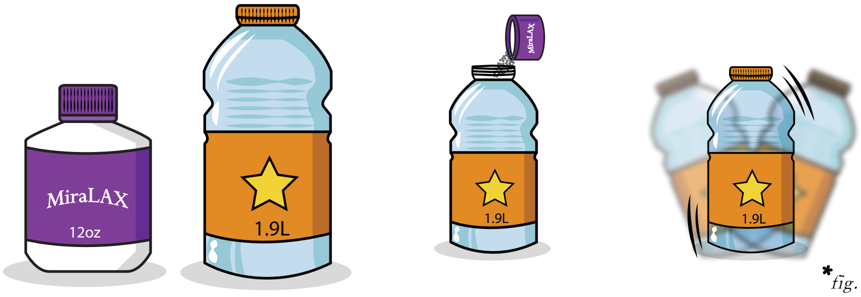

*fig. 2

If you have any problems with the preparation, please call the office at

718 270 4714

After office hours, can page Pediatric GI Fellow on call at

917 760 1157

After the Miralax mixture is gone, take the 3 Bisacodyl (Dulcolax) tablets with 8-12 oz of clear liquid.*

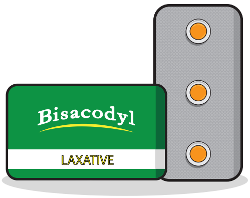

**fig. 3*

Drink clear liquids until midnight- then nothing by mouth. Not even gum or hard candy

AFTER THE PROCEDURE:

Immediately following the procedure, the doctor will come and speak with you about his or her observations during the test. Additional information will be obtained from the biopsy report, which will be available in about a week. You may call the office in a week to speak with the physician who requested that you child have the colonoscopy.

After the procedure your child will be taken to the Recovery Room, where you are invited to stay with them. After your child is awake and has had something to drink, they will be discharged home.

At home your child may participate in quiet, supervised activities, such as reading and watching TV. It is not uncommon for children to take frequent naps after anesthesia. Your child may experience a slight sore throat after the procedure, which should pass quickly.
Cold beverages or throat lozenges may be helpful. Some children experience nausea and vomiting due to the anesthesia.
Do not be alarmed if you notice flecks of blood in the vomit — this is from the biopsies and will stop within 24 hours. We advise first giving clear liquids such as juice, soda or Pedialyte. If these are tolerated, resume their usual diet. Depending on how your child is feeling, he or she may resume their normal activities and return to school on the following day.

DOWNSTATE MEDICAL CENTER | PEDIATRICS SUNY

If you need to cancel on the day of the procedure, or for directions on the day of the procedure, call the operating room:

718 270 1621

Please arrive on time. Allow extra time in your trip for weather, traffic and parking.

Check in at the registration desk at the ambulatory surgery.

PROCEDURE INFORMATION

PATIENT NAME:

DATE OF BIRTH:

PROCEDURE:

DATE OF PROCEDURE:

LOCATION:

SUNY DOWNSTATE MEDICAL CENTER.
445 LENOX ROAD
BROOKLYN NY 11203
2ND FLOOR, AMBULATORY SURGERY (NEAR RADIOLOGY)
RM 2-553

PRE-OPERATORY LIST:

- -CBC ☐
- -CMP ☐
- -ESR ☐
- -CRP ☐
- -COAG ☐

- -STOOL STUDIES
  - -stool culture ☐
  - -c. diff ☐
  - -o/p: ☐
- -FECAL CALPROTEIN ☐
- -N/A ☐
- -OTHER:____________________

If you have questions or to cancel/rescheudule your procedure call:
718 270 1621
